# Autologous bone marrow stem cell transplantation for patients undergoing coronary artery bypass grafting: a meta-analysis of 22 randomized controlled trials

**DOI:** 10.1186/s13019-022-01838-2

**Published:** 2022-06-25

**Authors:** Juelin Song, Kang He, Jianglong Hou

**Affiliations:** Department of Cardiovascular Surgery, West China Hospital, Sichuan University, Chengdu, 610041 Sichuan China

**Keywords:** Bone marrow, Stem cell, Coronary artery bypass, Meta‑analysis, Randomized controlled trial

## Abstract

**Background:**

Although the safety and feasibility of coronary artery bypass grafting (CABG) and bone marrow stem cell (BMSC) transplantation have been established, the effectiveness of this approach compared with CABG alone remains controversial. The aim of this updated meta-analysis of randomized controlled trials was to evaluate the efficacy of this procedure.

**Methods:**

A random-effects meta-analysis was conducted using studies sourced from the PubMed, Embase, and Cochrane literature databases to compare patients who received isolated CABG (CABG group) and BMSC transplantation with CABG (BMSC group). 22 studies were included.

**Results:**

A total of 22 relevant publications with 820 patients were included. 432 patients received BMSC transplantation with CABG and 388 patients received isolated CABG. Compared with the CABG group, the BMSC transplantation group exhibited an improvement in the left ventricular (LV) ejection fraction (mean difference (MD) = 3.87%; 95% confidence interval (CI): 1.93–5.80%; *P* < 0.001).

**Conclusion:**

The present evidence suggests that autologous BMSC transplantation for patients undergoing CABG appears to be associated with an improvement in LV function compared with CABG alone. However, heterogeneity in the data suggests that patients respond differently to this therapy. Further research is needed to understand these differences.

**Supplementary Information:**

The online version contains supplementary material available at 10.1186/s13019-022-01838-2.

## Introduction

Ischemic heart disease (IHD) remains one of the leading causes of morbidity and mortality worldwide, imposing both economic and healthy burdens on either developed and developing countries [1–3]. Myocardial ischemia often results in irreversible loss of viable myocardium and replacement by noncontractile scar tissue, leading to the impairment of left ventricular and cardiac dysfunction. Despite the advances in revascularization techniques and pharmacological treatment, patients with poor cardiac function struggle to achieve desirable outcomes. The aim of bone marrow stem cells (BMSCs) therapy is to repair damaged myocardium, prevent ventricular remodeling, and improve overall cardiac function [1].

Bone marrow cells are the first choice for bypass combined stem cell transplantation because they are autologous and readily available. The efficacy of CABG combined with BMSC transplantation remains controversial. Studies have shown that CABG combined with BMSC transplantation is beneficial to cardiac function without adverse reactions, and is a safe and feasible clinical adjuvant therapy [4–14]. However, other studies have reported no effect of CABG combined with BMSC transplantation on overall left ventricular function and clinical symptoms [13, 15–24]. Since the publication of these meta-analyses, several new randomized controlled trials (RCTs) have been published [11, 14, 22–24]. The purpose of this meta-analysis was to reevaluate the efficacy of CABG combined with BMSC transplantation by incorporating updated RCTs results.

## Methods

### Search strategy

The meta-analysis was performed following the Preferred Reporting Items for Systematic Reviews and Meta-analyses (PRISMA) guidelines [25] and was registered in the International Prospective Register of Systematic Reviews (PROSPERO) database (no. CRD42021276095). The PubMed, Cochrane Library and EMBASE were searched from inception to August 16, 2021. Detailed search strategies are shown in Additional file 1: Tables S1, Additional file 2: Table S2, Additional file 3: Table S3. The studies were not restricted by language, date of publication, or setting. To enhance detection, the reference lists of all selected published articles, relevant meta-analyses, systematic reviews, and editorials were hand-searched for other relevant articles.

### Selection criteria

Studies were included based on the following criteria: (1) RCTs comparing CABG in combination with BMSC transplantation and CABG alone for IHD; (2) follow-up for at least 3 months after stem cell therapy. The exclusion criteria were as follows: (1) catheter-based stem cell injection methods; (2) the published data did not include LVEF.

### Quality assessment

The quality of the selected RCTs was independently assessed by 2 reviewers (J. S. and K. H.) according to the Cochrane risk of bias criteria [26], with each quality item classified as low risk, high risk, and unclear risk. The 6 items used to evaluate bias in each trial included random sequence generation, allocation concealment, blinding of participants and personnel, blinding of outcome assessment, incomplete outcome data and selective reporting.

### Data extraction and outcomes

Two reviewers (J. S. and K. H.) extracted the following relevant data from each study independently: First author; year of publication; country of origin; study population, including BMSC and CABG group; follow-up time; participant characteristics, including age and sex; type of stem cells; the dose of stem cells; route of stem cell administration; treatment of CABG group; outcome measurement method; LV ejection fraction (LVEF), including baseline (LVEF_baseline_), follow-up (LVEF_follow-up_), and LVEF change from baseline to follow-up for the BMSC (LVEF_BMSC change_) and CABG groups (LVEF_CABG change_), and similarly, related data of LV end‑diastolic volume (LVEDV), LV end‑diastolic volume index (LVEDVI), LV end‑systolic volume (LVESV), LV end‑systolic volume index (LVESVI), LV end‑systolic diameter (LVESD), LV end‑diastolic diameter (LVEDD), 6-min walk test (6MWT). Because magnetic resonance imaging (MRI) is more accurate than echocardiography [27], MRI data are preferred in statistical analysis. Any disagreements between the reviewers were resolved by attending to a consensus.

### Statistical analysis

All statistical analyses were implemented with Review Manager 5.4 (RevMan, The Nordic Cochrane Centre, The Cochrane Collaboration, 2020) and Stata version 16.0 (StataCorp, College Station, TX, USA). A meta-analysis was performed to calculate the mean difference (MD) LVEF_change_ (MD LVEF_change_ = LVEF_BMSC change_-LVEF_CABG change_, LVEF_BMSC change_ = LVEF_BMSC follow-up_-LVEF_BMSC baseline_, LVEF_CABG change_ = LVEF_CABG follow-up_-LVEF_CABG baseline_), and similarly, the MD LVEDV_change_, MD LVEDVI_change_, MD LVESV_change_, MD LVESVI_change,_ MD LVESD_change_, MD LVEDD_change_, 6MWT_change_ as well as their 95% confidence intervals (CIs).

Most studies reported the mean and standard deviation (SD). In three studies [20, 21, 24], LV volume and ejection fraction values were expressed as mean and standard error (SE). In one study [24], the distance of 6MWT was expressed as mean and SE. The SD was calculated by the formula ${\text{SD}} = {\text{SE}} \times \surd {\text{n}}$, where n is the sample size. In two studies [7, 19], the LV volume and ejection fraction values were expressed as the median and interquartile range. In two studies [7, 18], distance of 6MWT was expressed as the median and interquartile range. Median and interquartile range were converted into the mean by the method introduced by Luo et al. [28] and converted into the SD by the method introduced by Wan et al. [29].

In addition, some studies [4, 6, 8–11, 13, 18, 30] did not directly report the mean and SD of LVEF_BMSC change_ and LVEF_CABG change_. The mean of the LVEF_BMSC change_ and LVEF_CABG change_ can be calculated by the difference between the means of the LVEF_baseline_ and LVEF_follow-up_. The SD of LVEF_BMSC change_ and LVEF_CABG change_ was calculated by the following formula:${\text{SD}}_{{{\text{change}}}} = \surd \left( {{\text{SD}}_{{{\text{baseline}}}}^{2} + {\text{SD}}_{{\text{follow - up}}}^{2} - 2 \times {\text{Corr}} \times {\text{SD}}_{{{\text{baseline}}}} \times {\text{SD}}_{{\text{follow - up}}} } \right)$. The SD of LVEF_BMSC change_ and LVEF_CABG change_ in the study by Hendrikx et al. [15] were used to calculate the Corr values by using the following formula: ${\text{Corr}} = \frac{{{\text{SD}}_{{{\text{baseline}}}}^{2} + {\text{SD}}_{{\text{follow - up}}}^{2} - {\text{SD}}_{{{\text{change}}}}^{2} }}{{2 \times {\text{SD}}_{{{\text{baseline}}}} \times {\text{SD}}_{{\text{follow - up}}} }}$. The Corr value of the BMSC group and CABG group was calculated to be 0.6. The mean and SD of the LV volume change values were calculated in the same manner.

A random-effects model was used to pool the data and I^2^ statistics were used to assess statistical heterogeneity between summary data. All tests were two-tailed and *P* < 0.05 was considered to indicate a statistically significant difference.

To evaluate whether the effectiveness of CABG combined with BMSC transplantation in IHD patients was influenced by the clinical characteristics, subgroup analyses were performed based on (1) follow-up time (> 6 or ≤ 6 months); (2) method to determine the outcome measure [echocardiography (ECHO), MRI OR single-photon emission computed tomography (SPECT)]; (3) type of stem cells [bone marrow mononuclear cells (BMMNCs), bone marrow cells(BMCs) or other selected cell populations (CD133 + and CD34 + cells)]; (4) route of injection [intramyocardial (IM) or intracoronary (IC)]; (5) dose of stem cells [≥ 10^8^ or < 10^8^ cells (10^8^ was the median number of BMSCs injected)]; (6) baseline LVEF < 30 or ≥ 30%. Analyses were performed to evaluate whether the differences between the subgroups were statistically significant. Leave-one-out sensitivity analysis of the primary outcome LVEF was performed.

## Results

### Search results

**Fig. 1:**
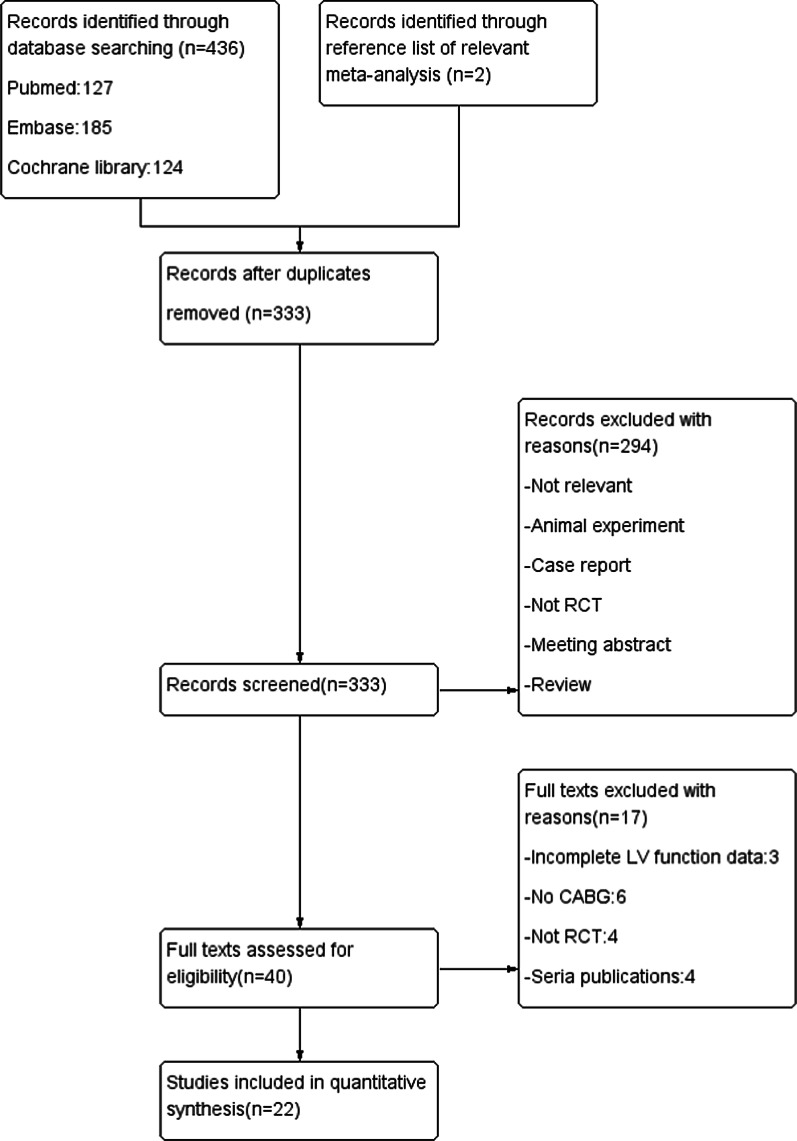
The flow diagram for study search process

A total of 436 studies were identified from the electronic database search. Finally, 20 independent RCTs were included in the analysis according to our search strategy. There were 2 relevant randomized controlled trials identified after researching the reference list of relevant literature (n = 2). These 4 literature [13, 31–33] reports one trial. The final analysis included 22 independent RCTs [4–24, 30]. A flow chart for the study selection process is presented in Fig. 1.

### Study characteristics

A total of 22 studies were included in the present meta-analysis. A total of 914 participants were assessed for the baseline of the studies. The ‘BMSC group’ (n = 484) included participants who had received CABG combined with BMSC transplantation, while the ‘CABG group’ (n = 430) included patients who had only received CABG. The follow-up period was range from 4 to 60 months. The mean age of the participants ranged from 51.7 to 66.8 years, and the percentage of male patients ranged from 70 to 100%. A total of 7 studies were performed in China, 3 in Germany and 1 each in Argentina, UK, Canada, Serbia, Spain, Finland, France, Iran, Netherlands, Indonesia, Turkey and Belgium. The baseline characteristics of the included studies are summarized in Table 1.

**Table 1:** Baseline characteristics of the included studies

Fist author (year)	Country	Sample size (n)	BMSC group (n)	CABG group (n)	Follow-up (months)	Age (years)		Sex, (male/female)		Type of stem cells	Dose of stem cells	Route of cell administration	Treatment of control group	Method for determining outcome measure
						BMSC Group	CABG Group	BMSC Group	CABG Group					
Patel et al. (2005)	Argentina	20	10	10	6	64.8 ± 3.9	63.6 ± 4.9	8/2	8/2	CD 34 +	22.0 × 10^6(median)	IM	off-pump CABG-only	ECHO
Hendrikx et al. (2006)	Belgium	20	10	10	4	63.2 ± 8.5	66.8 ± 9.2	10/0	7/3	BMC	60.25 ± 31.35 × 10^6	IM	CABG-only	MRI
Stamm et al. (2007)	Germany	40	20	20	12	62 ± 10.2	63.5 ± 8.4	15/5	16/4	CD 133 +	5.80 ± 10^6(median)(range 1.08 × 10^6 to 8.35 × 10^7)	IM	CABG-only	ECHO
Ang et al. (2008)	UK	62	21/21^a^	20	6	64.7 ± 8.7/62.1 ± 8.7	61.3 ± 8.3	15/6,19/2	18/2	BMC	84 ± 56 × 10^6/115 ± 73 × 10^6	IM/IC	CABG-only	MRI
Zhao et al. (2008)	China	36	18	18	6	60.3 ± 10.4	59.1 ± 15.7	15/3	15/3	BMMNC	6.59 ± 5.12 × 10^8	IM	CABG + placebo	ECHO
Hu et al. (2011)	China	60	31	29	6	56.61 ± 9.72	58.27 ± 8.86	NA	NA	BMMNC	13.17 ± 10.66 × 10^7	IC	CABG + placebo	MRI
Maureira et al. (2012)	France	14	7	7	6	58 ± 10	57 ± 10	7/0	6/1	BMMNC	NA	IM	CABG only	MRI
Lu et al. (2013)	China	50	25	25	12	58.0 ± 7.8	57.0 ± 8.3	22/3	24/1	BMMNC	13.38 ± 8.14 × 10^7	IC	CABG + placebo	MRI
Nasseri et al. (2014)	Germany	60	30	30	6	61.9 ± 7.3	62.7 ± 10.6	28/2	29/1	CD 133 +	5.1 ± 1.017 × 10^6(median)(IQR 3.0 × 10^6 to 9.1 × 10^6)	IM	CABG + placebo	MRI
Pätilä et al. (2014)	Finland	39	20	19	12	65(57–73)(median)	64(58–70)(median)	19/1	18/1	BMMNC	8.4 × 10^8(median)(IQR 5.2 × 10^8 to 13.5 × 10^8)	IM	CABG + placebo	MRI
Trifunović et al. (2015)	Serbia	30	15	15	60(median)	53.8 ± 10.1	60 ± 6.8	14/1	14/1	BMMNC	70.7 ± 32.4 × 10^6	IM	CABG-only	ECHO
Wang et al. (2015)	China	90	45	45	6	61.4 ± 7.45	62.9 ± 6.93	37/8	35/10	BMC	5.21 ± 0.44 × 10^8	IM	CABG + placebo	ECHO
Noiseux et al. (2016)	Canada	33	19	14	6	66.4 ± 6.5	63.1 ± 7.2	17/2	13/1	CD 133 +	6.5 ± 3.1 × 10^6	IM	CABG + placebo	MRI
Wang et al. (2016)	China	33	17	16	24	65.6 ± 3.97	65.5 ± 5.6	NA	NA	BMMNC	98.5 ± 48.3 × 10^6	IM	CABG-only	ECHO
Lu et al. (2017)	China	40	20	20	24	51.7 ± 2.5	52.6 ± 3.8	8/12	12/8	CD 34 +	NA	IM	CABG-only	ECHO
Steinhoff et al. (2017)	Germany	58	28	30	6	64.0 ± 7.20	63.6 ± 7.75	26/2	26/4	CD 133 +	2.29 ± 1.42 × 10^6	IM	CABG + placebo	MRI
Laguna et al. (2018)	Spain	17	8	9	9	62.63 ± 8.35	64.78 ± 11.48	7/1	8/1	BMMNC	NA	IM	CABG + placebo	ECHO
Naseri et al. (2018)	Iran	77	21/30^b^	26	18	53.14 ± 8.56/51.45 ± 7.49	55.50 ± 8.54	19/2,27/3	23/3	MNC/CD 133 +	8.19 ± 4.26 × 10^6/564.63 ± 69.35 × 10^6	IM	CABG + placebo	SPECT
Qi et al. (2018)	China	42	24	18	12	57.88 ± 8.52	56.56 ± 9.09	23/1	17/1	BMMNC	13.28 ± 9.41 × 10^7	IC	CABG + placebo	MRI
Mann et al. (2019)	Netherlands	39	19	20	12	65 ± 7	65 ± 8	19/0	18/2	BMMNC	100 × 10^6	IM	CABG + placebo	SPECT
Soetisna et al. (2020)	Indonesia	26	13	13	6	54.61 ± 8.07	57.46 ± 6.33	12/1	12/1	CD133 +	NA	IM	CABG-only	MRI
Ulus et al. (2020)	Turkey	28	12	16	12	56.9 ± 1.5(SE)	65.3 ± 1.7(SE)	12/0	16/0	BMMNC	70 × 10^7	IM	CABG-only	MRI

Values are expressed as the mean ± standard deviation

^a^Three arms for the study: IM BMSC injection vs IC BMSC injection vs CABG alone

^b^Three arms for the study: MNC injection vs CD 133 + injection vs CABG alone

### Risk of bias assessment

**Fig. 2:**
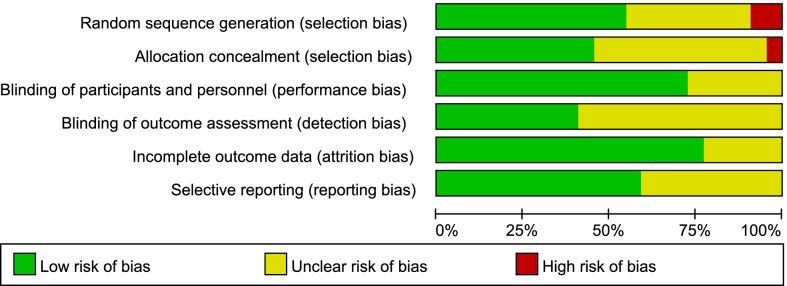
Risk of bias graph

**Fig. 3:**
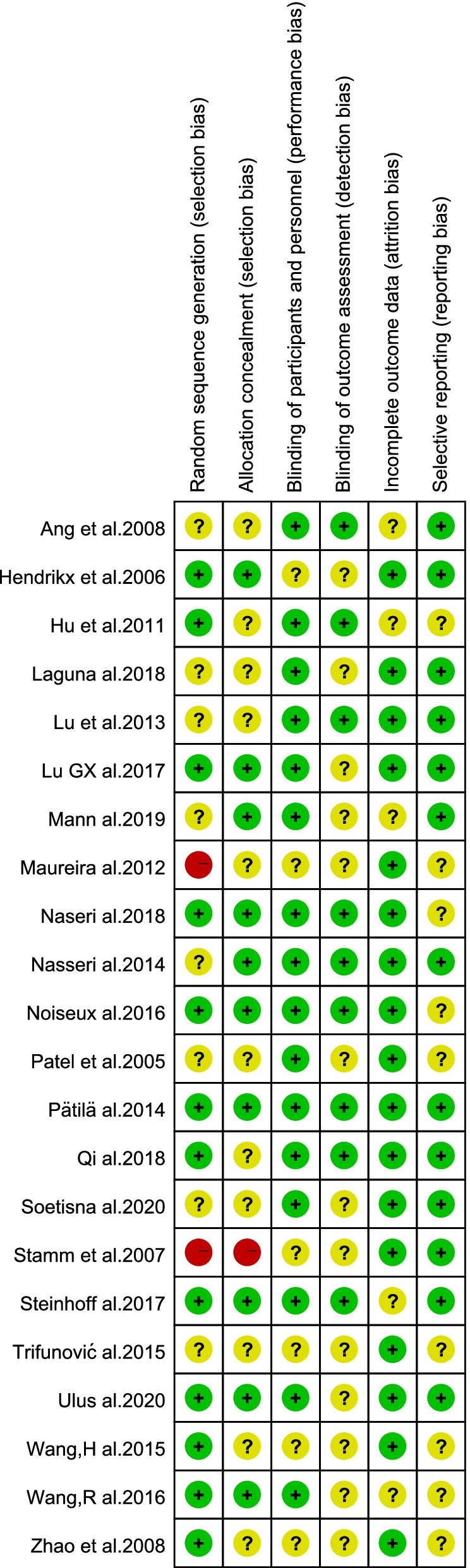
Risk of bias summary

Of the 22 studies, 12 (54.5%) adequately generated their randomization sequence, 10 (45.4%) concealed allocation, 16 (72.7%) blinded participants and personnel, 9 (40.9%) blinded outcome assessment,17(77.3%) studies had a low risk of incomplete outcome data and 13(59.1%) studies had a low risk of selective reporting. The detailed information on the risk of bias is provided in Figs. 2 and 3.

### LV function

**Fig. 4:**
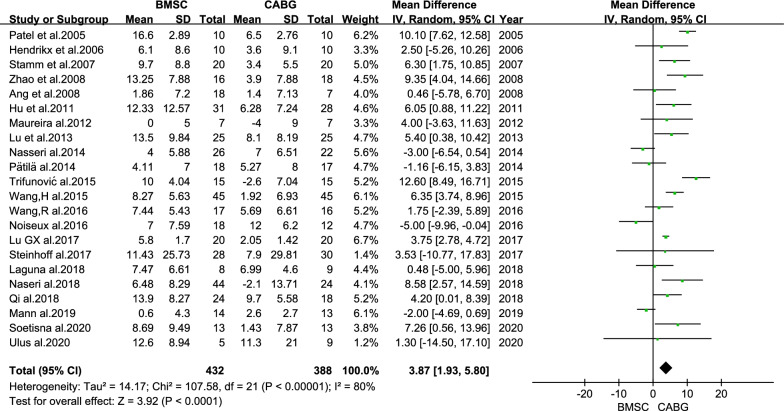
Forest plot of the difference in the change from baseline in the LVEF between the BMSC and CABG groups

LVEF has been reported in all 22 studies, including a total of 820 participants. The difference in the change of the LVEF between the BMSC and CABG groups was statistically significant (MD = 3.87%; 95% CI: 1.93 to 5.80%; *P* < 0.001; Fig. 4).

**Fig. 5:**
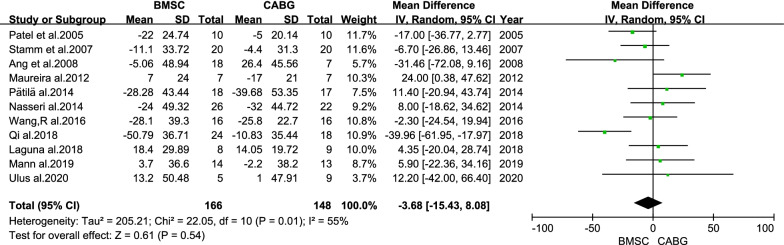
Forest plot of the difference in the change from baseline in the LVEDV between the BMSC and CABG groups

**Fig. 6:**
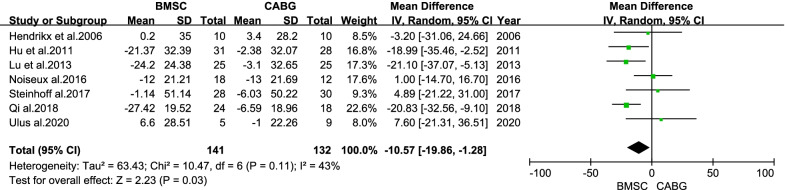
Forest plot of the difference in the change from baseline in the LVEDVI between the BMSC and CABG groups

There was no statistical difference in the overall change of LVEDV from baseline to follow-up between the BMSC and CABG groups (MD = −3.68 ml; 95% CI: −15.43 to 8.08 ml; *P* = 0.54; Fig. 5). The difference in the change of the LVEDVI between the BMSC and CABG groups was statistically significant (MD = −10.57 ml/m^2^; 95% CI: −19.86 to −1.28 ml/m^2^; *P* = 0.03; Fig. 6).

**Fig. 7:**
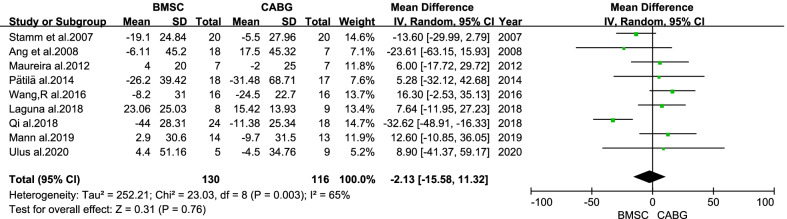
Forest plot of the difference in the change from baseline in the LVESV between the BMSC and CABG groups

**Fig. 8:**
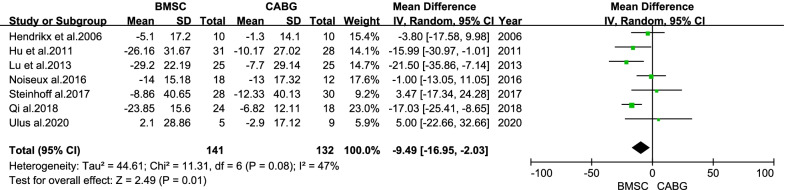
Forest plot of the difference in the change from baseline in the LVESVI between the BMSC and CABG groups

There was no statistical difference in the overall change of LVESV from baseline to follow-up between the BMSC and CABG groups (MD = −2.13 ml; 95% CI: −15.58 to 11.32 ml; *P* = 0.76; Fig. 7). There was a statistical difference in the overall change of LVESVI from baseline to follow-up between the BMSC and CABG groups (MD = -9.49 ml/m^2^; 95% CI: −16.95 to −2.03 ml/m^2^; *P* = 0.01; Fig. 8).

**Fig. 9:**
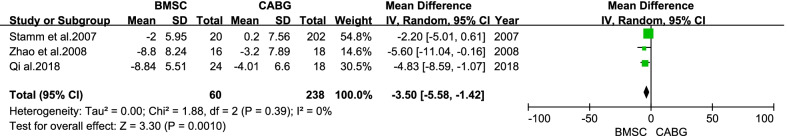
Forest plot of the difference in the change from baseline in the LVESD between the BMSC and CABG groups

**Fig. 10:**
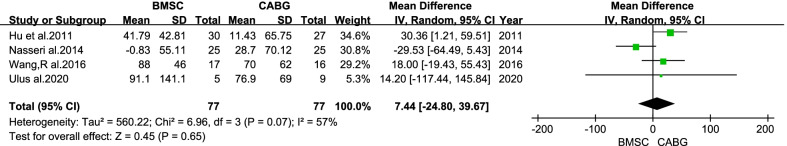
Forest plot of the difference in the change from baseline in the LVEDD between the BMSC and CABG groups

A total of 3 studies with 298 participants reported a change in LVESD after the treatment. There was a statistical difference in the overall change of LVESD from baseline to follow-up between the BMSC and CABG groups (MD = −3.50 mm; 95% CI: −5.58 to −1.42 mm; *P* = 0.001; Fig. 9). A total of 4 studies with 164 participants reported a change in LVEDD after the treatment. There was no statistical difference in the overall change of LVEDD from baseline to follow-up between the BMSC and CABG groups (MD = −2.49 mm; 95% CI: −7.27 to 2.28 mm; *P* = 0.31; Fig. 10).

### 6MWT

**Fig. 11:**
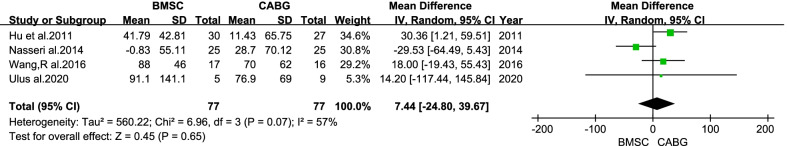
Forest plot of the difference in the change from baseline in the 6MWT between the BMSC and CABG groups

A total of 4 studies with 154 participants reported a change in 6MWT after the treatment. There was no statistical difference in the overall change of 6MWT from baseline to follow-up between the BMSC and CABG groups (MD = 7.44 m; 95% CI: −24.80 to 39.67 m; *P* = 0.65; Fig. 11).

### Publication bias

To exclude potential publication bias, funnel plots for publication bias were performed. No publication bias was evident for the studies included in the LVEF, LVEDV, LVESV, LVESD, LVEDD and 6MWT meta-analysis. But publication bias may exist for the studies included in the LVEDVI and LVESVI according to funnel plots.

### Subgroup analysis and sensitivity analysis of LVEF

No statistical differences have been found within subgroups based on follow-up period, type of stem cells, route of cell administration, dose of stem cells and baseline LVEF, except the subgroups of measurement method for the LVEF (ECHO, SPECT or MRI) (*P* = 0.05; Table 2). Leave-one-out sensitivity analysis indicated that the results were not markedly affected by the exclusion.

**Table 2:** Subgroup analysis of LVEF change

Variable	No. of trials	MD[95% CI]	Heterogeneity (I^2^) (%)	*P*-value
**Follow-up for examining LVEF (months)**				
> 6	10	3.54 [0.91, 6.17]	79	0.79
≤ 6	12	4.10 [0.92, 7.28]	81	
**Method of measurement**				
ECHO	8	6.39 [3.71, 9.08]	84	0.05
MRI	12	1.80 [−0.77, 4.36]	55	
SPECT	2	2.93 [−7.41,13.28]	90	
**Type of stem cells**				
BMMNC/BMC	14	3.84 [1.28, 6.40]	75	0.82
CD133 + /CD34 +	7	3.29 [−0.58, 7.16]	89	
**Route of cell administration**				
IM	18	3.80 [1.53, 6.06]	84	0.48
IC	3	5.07 [2.34, 7.80]	0	
**Amount of stem cells administered**				
≥ 10^8^	8	3.78 [0.52, 7.03]	77	0.57
< 10^8^	8	5.34 [1.05, 9.63]	84	
**Baseline LVEF (%)**				
< 30	6	3.97 [−0.68, 8.62]	87	0.94
≥ 30	16	3.78 [1.58, 5.99]	77	

## Discussion

In this meta-analysis, CABG combined with BMSC transplantation showed an improved cardiac function in patients with IHD compared with CABG alone. The change of LVEF from baseline to follow-up in the BMSC group increased by 3.87% (CI: 1.93–5.80%) compared with that in the CABG group. But the results were highly heterogeneous (I^2^ = 80%). A detailed subgroup analysis was performed to explore differences in LVEF change and revealed that these results were consistent regardless of the follow-up time, type of stem cells, route of cell injection (IM or IC), dose of stem cells and baseline LVEF.

Subgroup analysis of LVEF measurements (echocardiography, SPECT, or MRI) showed that the choice of method influenced the determined effectiveness of CABG combined with BMSC transplantation in IHD patients. Method of LVEF measurements was revealed as a significant factor contributing to the heterogeneity of the results. In addition, subgroup analysis of echocardiographic tests demonstrated higher values of LVEF improvement but poor homogeneous results. However, subgroup analysis of MRI did not show any significant improvement of LVEF and more homogeneous results. Echocardiography, SPECT and MRI have important diagnostic value in assessing cardiac function. Nonetheless, echocardiographic measurements are affected by the ultrasonographer, whereas MRI and SPECT are more reliable and accurate for measuring cardiac function in IHD patients. The source of heterogeneity in these results cannot be identified sufficiently. Although the subgroup analysis showed the method of LVEF and SPECT assessment as significant factors, this finding could not clinically explain the differences in the outcomes reported by different trials. The high SD values in some trials may demonstrate that the cause of the different outcomes reported by the trials might be due to variation in patient response to BMSC transplantation.

Subgroup analysis suggested that the use of BMMNCs or BMCs may lead to a more pronounced improvement in LVEF compared to CD133 + or CD34 + cells, that 2 of the 7 studies that included CD133 + or CD34 + cells in the meta-analysis had an unfavorable MD, and 2 of the 10 studies using BMMNCs or BMCs had an unfavorable MD. But the study of Naseri et al. [12] suggested that CD133 + cells had slightly greater efficacy compared to BMMNCs. Naseri et al. [12] have commented that the heterogeneous population of the BMMNCs may affect homing of the desired cells and previous human studies have shown that intracoronary transplantation with a small concentration of bone marrow progenitor cells has a sevenfold higher homing ability compared to larger numbers of BMMNCs. Noiseux et al. [20] suggested that selected CD133+, CD34+ , CD45+ hematopoietic progenitor cells have vasculogenic properties that may improve perfusion in ischemic cardiomyopathy. However, results may be limited by the small sample size of groups treated with CD133+ or CD34+ cells and autologous cell agents are medical products characterized by the complexity of cell isolation protocols and cell product storage, and the methods used to evaluate the results may be inhomogeneous. These factors may affect the effectiveness of cell therapy in improving heart function. In addition, subgroup analysis of dose of stem cells demonstrated that the number of injected BMSCs was not a significant factor affecting the heterogeneity of the data, and the change of LVEF may be independent of the dose of BMSCs.

Subgroup analysis of IC injection demonstrated higher values of LVEF improvement but poor homogeneous results [MD 5.07% (2.34 to 7.80%), I^2^ = 0%], while MD of IM injection group is 3.80%(1.53 to 6.06, I^2^ = 84%). Hu et al. [7] have commented that stem cells in the process of operation were shipped to the myocardial, mainly around the infarction area, while a large number of transplanted cells by intramyocardial in situ, but a large number of cells during ischemia or infarction area reducing survival and impairing proliferation ability, in addition, the uneven distribution of delivery within myocardial cells, some parts need to cell therapy can't reach. In their study [7], the aorta was open 5 min after cell injection, extending the contact time between BMMNCs and small coronary vessels and enhancing the adhesion of BMMNCs, thereby reducing the number of BMMNCs washed out of the heart. They [7] hypothesized that in a cardiac arrest, capillaries dilate and blood vessel permeability increases, so transplanted cells attached to the blood vessel wall migrate easily to the myocardium. Naseri et al. [12] hold the opposite view that intramuscular injection was the most effective method because cells are more reliably located in the heart by direct visualization and delivery to the target site, while many of the injected cells deliver to the lungs or liver with intracoronary injections. More high-quality research is needed to determine which approach is better.

LVESV_change_ and LVEDV_change_ decreased in the BMSC group, but the difference was not statistically significant compared with the CABG group. Our meta-analysis demonstrated that there was a statistical difference in LVEDVI_change_ (MD = −10.57 ml/m^2^; 95% CI: −19.86 to −1.28 ml/m^2^; *P* = 0.03) and LVESVI_change_ (MD = −9.49 ml/m^2^; 95% CI: −16.95 to −2.03 ml/m^2^; *P* = 0.01) between the BMSC and CABG groups, while the meta-analysis of Wu et al. [1] with 14 RCTs revealed no statistically difference between two groups. These indexes are more reflective regarding the heart function compared with LVEDV and LVESV, as each individual's body surface area is different. This may be one of the reasons for the difference in LVEDV and LVESV not being statistically significant.

LVESD_change_ and LVEDD_change_ decreased in the BMSC group compared with the CABG group. There was a statistically difference in LVESD_change_ (MD = −3.50 mm; 95% CI: −5.58 to −1.42 mm; *P* = 0.001) and no statistically difference in LVEDD_change_ (MD = −2.49 mm; 95% CI: −7.27 to 2.28 mm; *P* = 0.31). This meant the BMSC group may benefit more than the CABG group in LVESD.

There are some points of view in previous studies. Wang et al. [30] have commented that paracrine effects of BMMNCs transplantation and the intervention time may play a key role in the outcome, the left ventricular remodeling is more likely to be prohibited and the left ventricular systolic function obtains the opportunity to improve steadily in the long term while transplanted at the acute myocardial infarction setting, limited reduction in MI size, short-term improvement in LV function, and disappearance of paracrine effects over time when BMMNCs is transplanted at old myocardial infarction setting in which the left ventricular remodeling has already developed and the paracrine effect of BMMNCs is mainly acting on the transitional zone of old myocardial infarction. Wang et al. [10] suggested that transplantation during off-pump coronary artery bypass grafts could reduce ischemia and reperfusion injury and restore vascular supply, thereby increasing stem cell survival rate and avoiding inflammation, loss of survival signal of extracellular matrix components and release of ischemic cardiac cytotoxic factors leading to high mortality of stem cells after transplantation.

BMSCs are an ideal cell resource for cell therapy. BMSCs are easy to harvest, and the biological characteristics are not affected after isolation. There are several important subclasses, such as endothelial progenitor cells, mesenchymal stem cells, and hemopoietic progenitor cells; each type may be capable of improving heart function [7].

The detailed mechanism of autologous bone marrow stem cell transplantation for patients undergoing coronary artery bypass grafting has not been fully elucidated. The role of CD133+ cells in reducing nonviable segments, improving LVEF and wall thickening is unclear. Naseri et al. [12] have commented that previous animal experiments have shown that the transplanted cells integrate into the new environment and form new vasculature and myocardium. Wang et al. [30] have commented that a series of experimental studies have shown that BMMNCs can express a large number of cytokines, prevent cardiomyocyte apoptosis, promote angiogenesis, and recruit endogenous stem cells for cell regeneration and fusion. Wang et al. [10] have suggested that the suppression of fibrosis and the improvement of ventricular remodeling induced by BMCs transplantation may play important roles in improving cardiac function. Lu et al. [11] have commented that autologous BMMNCs transplantation increased viable myocardium and improved microcirculation of infarcted myocardium. Previous animal studies have suggested that exogenous Shh protein may promote the improvement of cardiac function of CD34+ cells after bone marrow stem cell transplantation [11]. Previous studies have shown that erythropoietin combined with granulocyte-colony-stimulating factor can enhance vascular formation and reduce infarct area after bone marrow stem cell transplantation in myocardial infarction area by increasing endothelial progenitor cell mobilization and up-regulating vascular endothelial growth factor and other microenvironments [11].

Overall, the results of this meta-analysis should be interpreted with caution, especially the results of subgroup analyses, as the number of studies per subgroup is further reduced. Therefore, future meta-analyses must include more studies to obtain significant results.

## Limitations

While the results of this study seem promising for the efficacy of BMSC transplantation, there were also certain limitations: There was significant heterogeneity in the present meta-analysis. Follow-up work in most studies is relatively short, and the continued efficacy of BMSC transplantation in patients treated with CABG remains to be demonstrated.

## Conclusion

Based on current evidence, autologous BMSC transplantation in patients receiving CABG appears to be associated with improved LV function, and this improvement is beyond that achieved by CABG alone. BMSC transplantation seems to be beneficial for patients receiving CABG. However, the differences in patients' responses to this treatment require further study. Future research should focus on patient profiling and response to treatment to identify the patient population that could benefit most from this approach and the mechanisms of action of BMSC transplantation.

## Supplementary Information

**Additional file 1: Table S1.** Search strategy in Pubmed.

**Additional file 2: Table S2.** Search strategy in Embase.

**Additional file 3: Table S3.** Search strategy in Cochrane library.

**Additional file 4:** Funnel plot.

**Additional file 5:** Subgroup analysis of LVEF (Forest plot and Funnel plot).

## Data Availability

All data generated or analysed during this study are included in this published article [and its additional files].

## Abbreviations

CABG: Coronary artery bypass grafting
BMSC: Bone marrow stem cell
LV: Left ventricular
MD: Mean difference
CI: Confidence interval
IHD: Ischemic heart disease
BMSCs: Bone marrow stem cells
RCTs: Randomized controlled trials
LVEF: LV ejection fraction
LVEDV: LV end‑diastolic volume
LVEDVI: LV end‑diastolic volume index
LVESV: LV end‑systolic volume
LVESVI: LV end‑systolic volume index
LVESD: LV end‑systolic diameter
LVEDD: LV end‑diastolic diameter
6MWT: 6-Minute walk test
MRI: Magnetic resonance imaging
SD: Standard deviation
SE: Standard error
ECHO: Echocardiography
SPECT: Single-photon emission computed tomography
BMMNCs: Bone marrow mononuclear cells
BMCs: Bone marrow cells
IM: Intramyocardial
IC: Intracoronary
