# Supplementary material for: Autologous bone marrow stem cell transplantation for patients undergoing coronary artery bypass grafting: a meta-analysis of 22 randomized controlled trials
Source: J Cardiothorac Surg. 2022 Jun 25;17:167. doi: 10.1186/s13019-022-01838-2 (PMC9233763; doi:10.1186/s13019-022-01838-2)
Supplement: Supplementary file 1 — Additional file 1: Table S1. Search strategy in Pubmed. [file 13019_2022_1838_MOESM1_ESM.docx]

| Procedure | Subject terms and free text terms used when retrieving literatures |
| --- | --- |
| #1 | "Stem Cells"[Mesh] |
| #2 | "Bone Marrow Transplantation"[Mesh] |
| #3 | "Coronary Artery Bypass"[Mesh] |
| #4 | ((Cell, Stem[Title/Abstract]) OR (Cells, Stem[Title/Abstract]) OR (Stem Cell[Title/Abstract]) OR (Progenitor Cells[Title/Abstract]) OR (Cell, Progenitor[Title/Abstract]) OR (Cells, Progenitor[Title/Abstract]) OR (Progenitor Cell[Title/Abstract]) OR (Mother Cells[Title/Abstract]) OR (Cell, Mother[Title/Abstract]) OR (Cells, Mother[Title/Abstract]) OR (Mother Cell[Title/Abstract]) OR (Colony-Forming Unit[Title/Abstract]) OR (Colony Forming Unit[Title/Abstract]) OR (Colony-Forming Units[Title/Abstract]) OR (Colony Forming Units[Title/Abstract])) |
| #5 | ((Grafting, Bone Marrow[Title/Abstract]) OR (Bone Marrow Grafting[Title/Abstract]) OR (Transplantation, Bone Marrow[Title/Abstract]) OR (Bone Marrow Cell Transplantation[Title/Abstract]) OR (Transplantation, Bone Marrow Cell[Title/Abstract])) |
| #6 | ((Artery Bypass, Coronary[Title/Abstract]) OR (Artery Bypasses, Coronary[Title/Abstract]) OR (Bypasses, Coronary Artery[Title/Abstract]) OR (Coronary Artery Bypasses[Title/Abstract]) OR (Coronary Artery Bypass Surgery[Title/Abstract]) OR (Bypass, Coronary Artery[Title/Abstract]) OR (Aortocoronary Bypass[Title/Abstract]) OR (Aortocoronary Bypasses[Title/Abstract]) OR (Bypass, Aortocoronary[Title/Abstract]) OR (Bypasses, Aortocoronary[Title/Abstract]) OR (Bypass Surgery, Coronary Artery[Title/Abstract]) OR (Coronary Artery Bypass Grafting[Title/Abstract])) |
| #7 | (clinical[tiab] AND trial[tiab]) OR "clinical trials as topic"[mesh] OR "clinical trial"[pt] OR random*[tiab] OR "random allocation"[mesh] OR "therapeutic use"[sh] |
| #8 | #1 OR #4 |
| #9 | #2 OR #5 |
| #10 | #3 OR #6 |
| #11 | # 8 OR #9 |
| #12 | #11 AND #10 |
| #13 | #12 AND #7 |

**Table S1** Search strategy in Pubmed
