# Supplementary material for: Autologous bone marrow stem cell transplantation for patients undergoing coronary artery bypass grafting: a meta-analysis of 22 randomized controlled trials
Source: J Cardiothorac Surg. 2022 Jun 25;17:167. doi: 10.1186/s13019-022-01838-2 (PMC9233763; doi:10.1186/s13019-022-01838-2)
Supplement: Supplementary file 2 — Additional file 2: Table S2. Search strategy in Embase. [file 13019_2022_1838_MOESM2_ESM.docx]

| Procedure | Subject terms and free text terms used when retrieving literatures |
| --- | --- |
| #1 | 'coronary artery surgery'/exp |
| #2 | 'bone marrow transplantation'/exp |
| #3 | 'stem cell'/exp |
| #4 | ('coronary artery surgery' OR 'coronary surgery' OR 'surgery, coronary artery'):ab,ti |
| #5 | ('bone marrow cell transfer' OR 'bone marrow graft' OR 'bone marrow grafting' OR 'bone marrow transfusion' OR 'bone marrow transplant' OR 'bone marrow transplantation' OR 'transplantation, bone marrow'):ab,ti |
| #6 | ('cell, stem' OR 'precursor cell' OR 'progenitor cell' OR 'stem cell' OR 'stem cells'):ab,ti |
| #7 | #1 OR #4 |
| #8 | #2 OR #5 |
| #9 | #3 OR #6 |
| #10 | #8 OR #9 |
| #11 | #7 AND #10 |
| #12 | 'randomized controlled trial'/exp OR 'controlled trial, randomized' OR 'randomised controlled study' OR 'randomised controlled trial' OR 'randomized controlled study' OR 'randomized controlled trial' OR 'trial, randomized controlled' |
| #13 | #11 AND #12 |

**Table S2** Search strategy in Embase
