# Supplementary material for: Autologous bone marrow stem cell transplantation for patients undergoing coronary artery bypass grafting: a meta-analysis of 22 randomized controlled trials
Source: J Cardiothorac Surg. 2022 Jun 25;17:167. doi: 10.1186/s13019-022-01838-2 (PMC9233763; doi:10.1186/s13019-022-01838-2)
Supplement: Supplementary file 3 — Additional file 3: Table S3. Search strategy in Cochrane library. [file 13019_2022_1838_MOESM3_ESM.docx]

| Procedure | Subject terms and free text terms used when retrieving literatures |
| --- | --- |
| #1 | MeSH descriptor: [Coronary Artery Bypass] explode all trees |
| #2 | MeSH descriptor: [Stem Cells] explode all trees |
| #3 | MeSH descriptor: [Bone Marrow Transplantation] explode all trees |
| #4 | (Coronary Artery Bypass Grafting):ti,ab,kw OR (Bypass, Coronary Artery):ti,ab,kw OR (Bypasses, Coronary Artery):ti,ab,kw OR (Artery Bypasses, Coronary):ti,ab,kw OR (Coronary Artery Bypass Surgery):ti,ab,kw OR (Bypasses, Aortocoronary) |
| #5 | (Coronary Artery Bypasses):ti,ab,kw OR (Bypass Surgery, Coronary Artery):ti,ab,kw OR (Bypass, Aortocoronary):ti,ab,kw OR (Artery Bypass, Coronary):ti,ab,kw OR (Aortocoronary Bypass):ti,ab,kw OR (Aortocoronary Bypasses):ti,ab,kw |
| #6 | (Stem Cell):ti,ab,kw OR (Cell, Stem):ti,ab,kw OR (Mother Cells):ti,ab,kw OR (Progenitor Cell):ti,ab,kw OR (Cells, Stem):ti,ab,kw OR (Cell, Progenitor):ti,ab,kw |
| #7 | (Mother Cell):ti,ab,kw OR (Cells, Mother):ti,ab,kw OR (Cells, Progenitor):ti,ab,kw OR (Progenitor Cells):ti,ab,kw OR (Cell, Mother):ti,ab,kw OR (Colony Forming Units):ti,ab,kw |
| #8 | (Colony-Forming Unit):ti,ab,kw OR (Colony-Forming Units):ti,ab,kw OR (Colony Forming Unit):ti,ab,kw |
| #9 | (Transplantation, Bone Marrow Cell):ti,ab,kw OR (Bone Marrow Cell Transplantation):ti,ab,kw OR (Grafting, Bone Marrow):ti,ab,kw OR (Transplantation, Bone Marrow):ti,ab,kw OR (Bone Marrow Grafting):ti,ab,kw |
| #10 | #1 OR #4 OR #5 |
| #11 | #2 OR #6 OR #7 OR #8 |
| #12 | #3 OR #9 |
| #13 | #11 OR #12 |
| #14 | #13 AND #10 |

**Table S3** Search strategy in Cochrane library
