# Supplementary material for: Autologous bone marrow stem cell transplantation for patients undergoing coronary artery bypass grafting: a meta-analysis of 22 randomized controlled trials
Source: J Cardiothorac Surg. 2022 Jun 25;17:167. doi: 10.1186/s13019-022-01838-2 (PMC9233763; doi:10.1186/s13019-022-01838-2)

**Funnel plot 4.** Funnel plot of the difference in the change from baseline in the LVEF between the BMSC and Control groups.


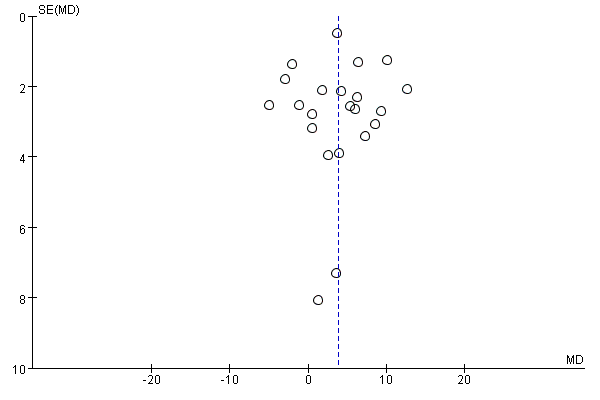


**Funnel plot 5.** Funnel plot of the difference in the change from baseline in the LVEDV between the BMSC and Control groups.


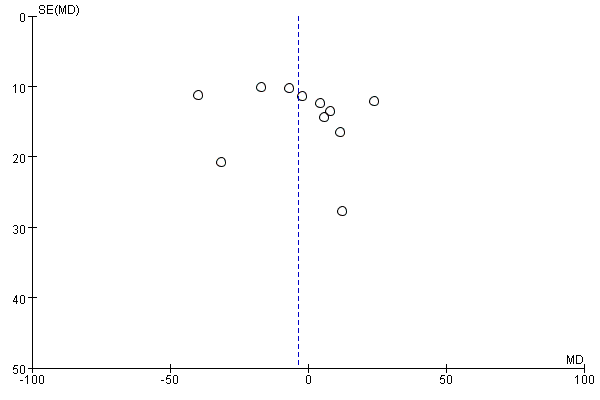


**Funnel plot 6.** Funnel plot of the difference in the change from baseline in the LVEDVI between the BMSC and Control groups.


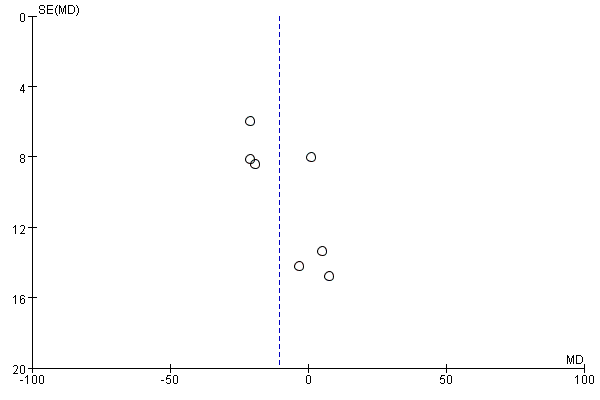


**Funnel plot 7.** Funnel plot of the difference in the change from baseline in the LVESV between the BMSC and Control groups.


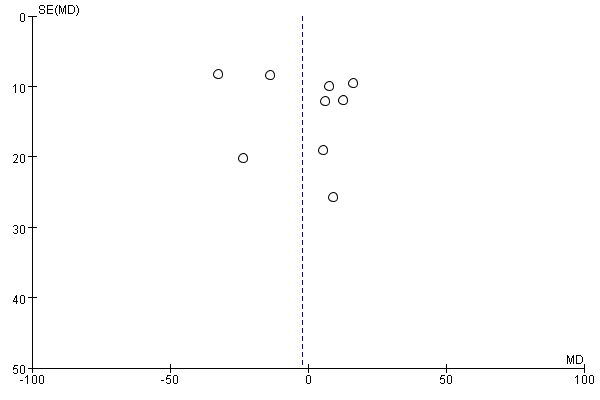


**Funnel plot 8.** Funnel plot of the difference in the change from baseline in the LVESVI between the BMSC and Control groups.


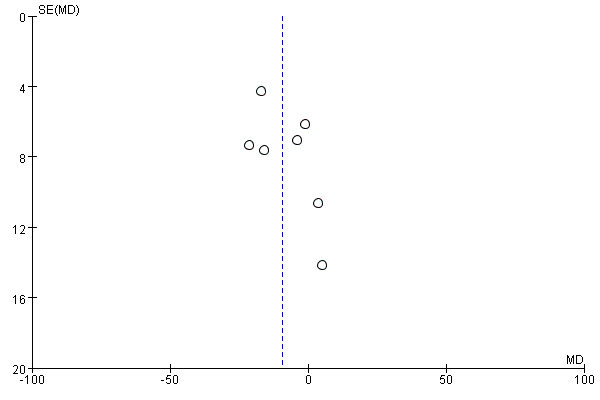


**Funnel plot 9.** Funnel plot of the difference in the change from baseline in the LVESD between the BMSC and Control groups.


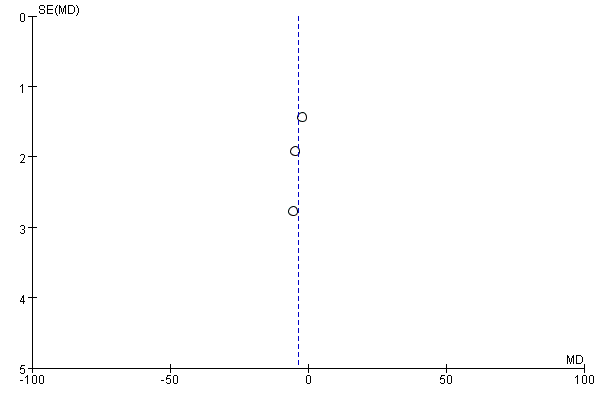


**Funnel plot 10.** Funnel plot of the difference in the change from baseline in the LVEDD between the BMSC and Control groups.


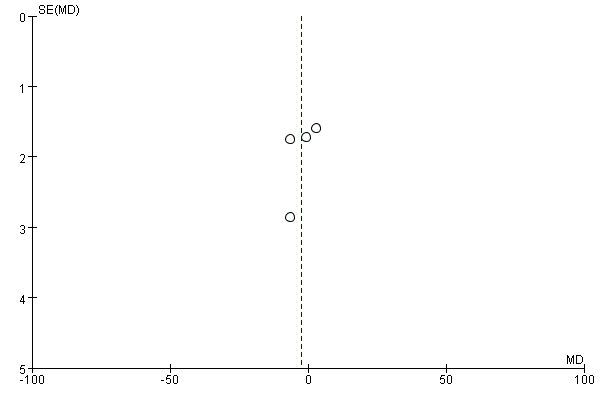


**Funnel plot 11.** Funnel plot of the difference in the change from baseline in the 6MWT between the BMSC and Control groups.


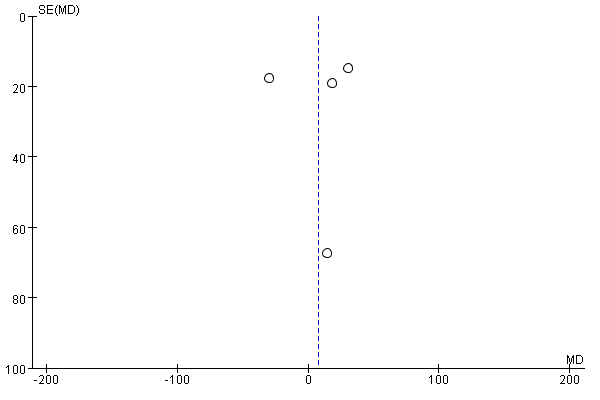

Supplement: Supplementary file 4 — Additional file 4: Funnel plot. [file 13019_2022_1838_MOESM4_ESM.docx]
