# Supplementary figures and images for: Autologous bone marrow stem cell transplantation for patients undergoing coronary artery bypass grafting: a meta-analysis of 22 randomized controlled trials
Source: J Cardiothorac Surg. 2022 Jun 25;17:167. doi: 10.1186/s13019-022-01838-2 (PMC9233763; doi:10.1186/s13019-022-01838-2)

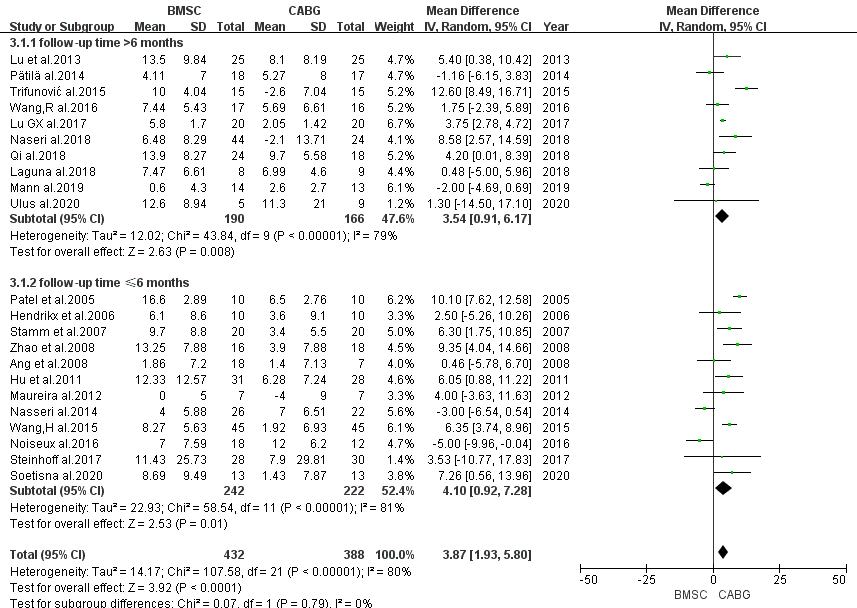

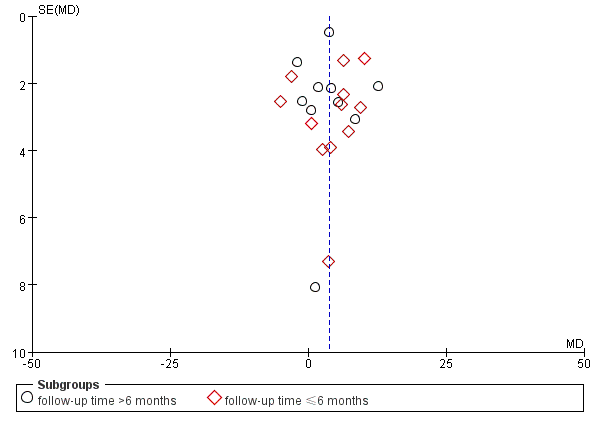


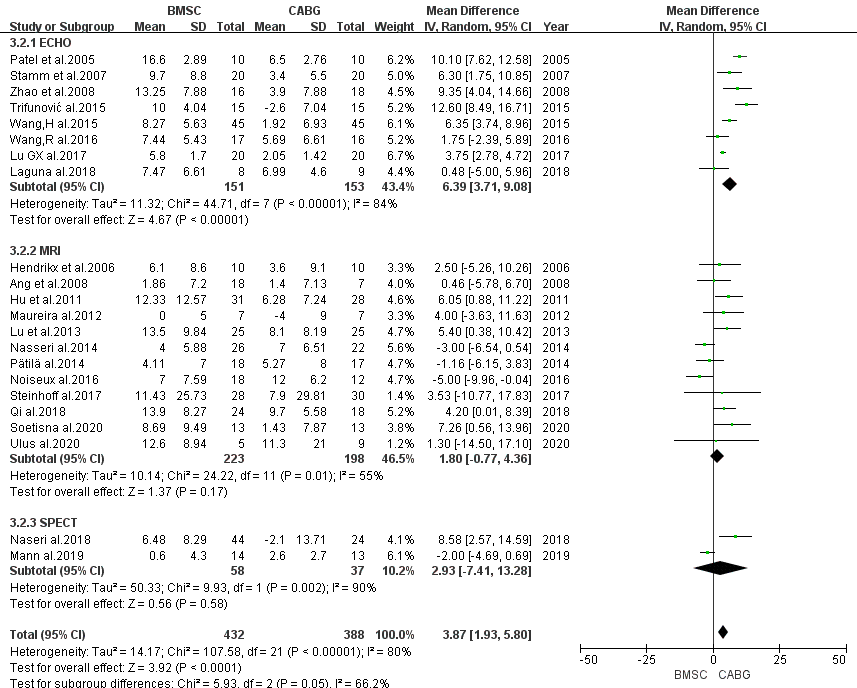

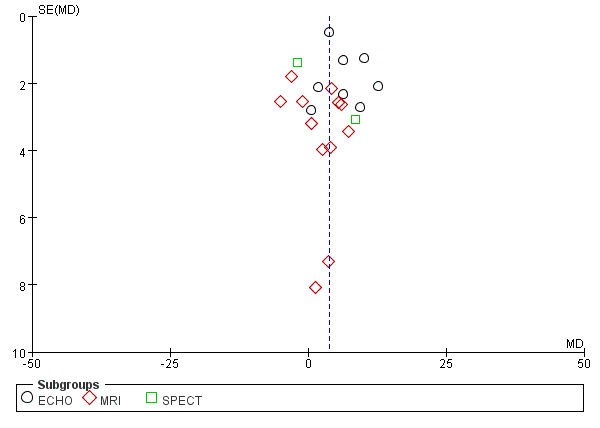


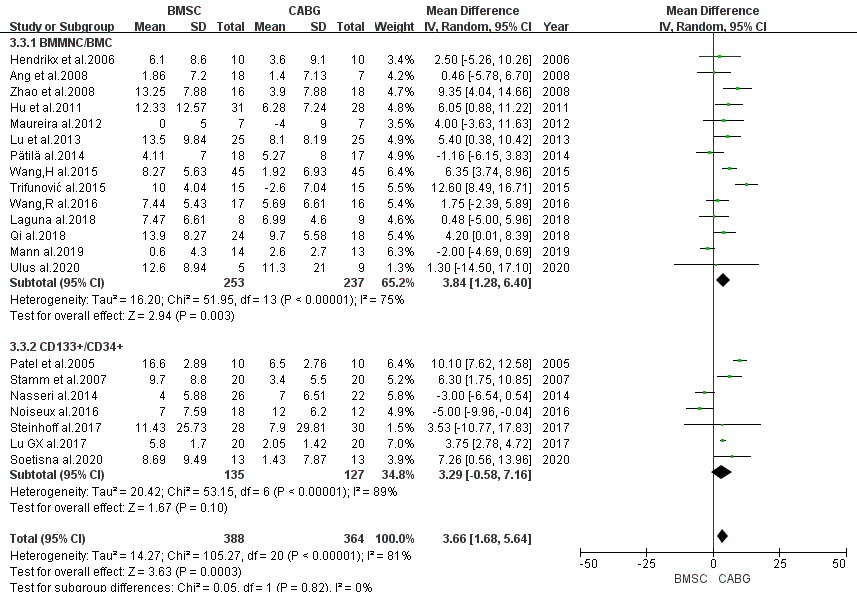

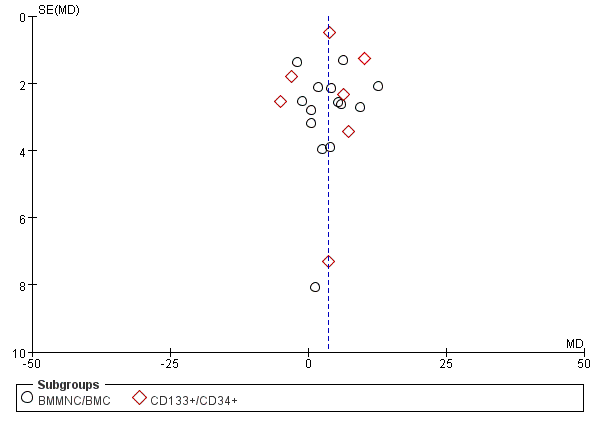


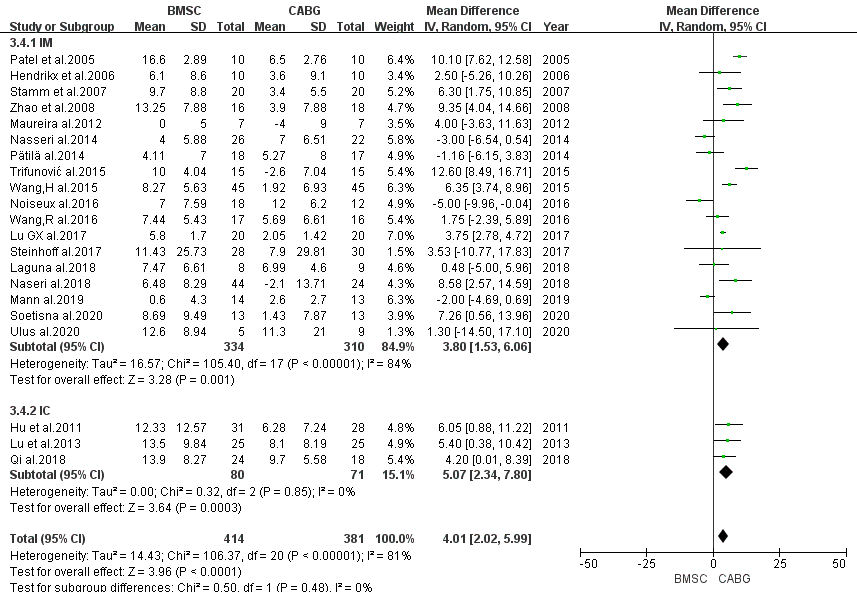


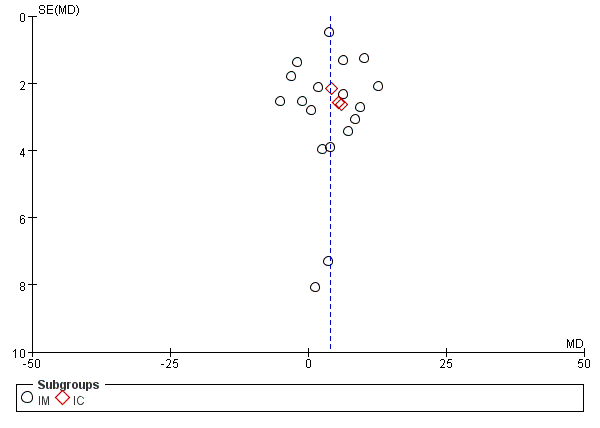


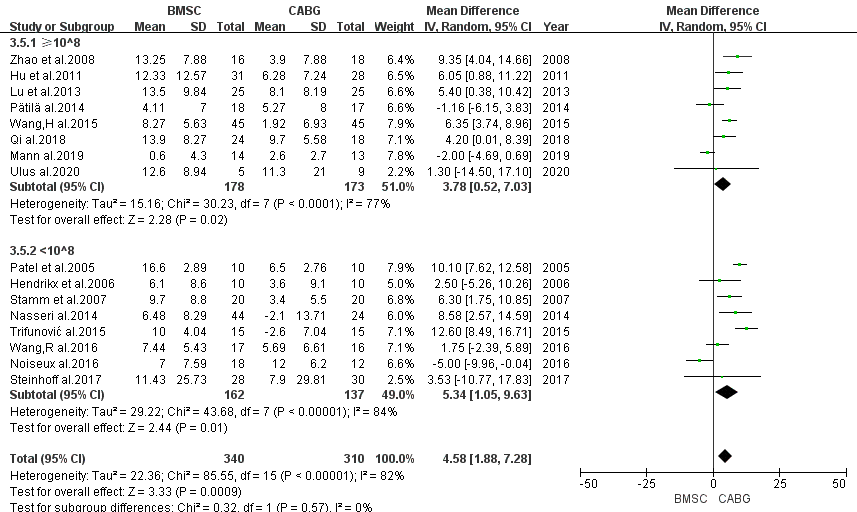

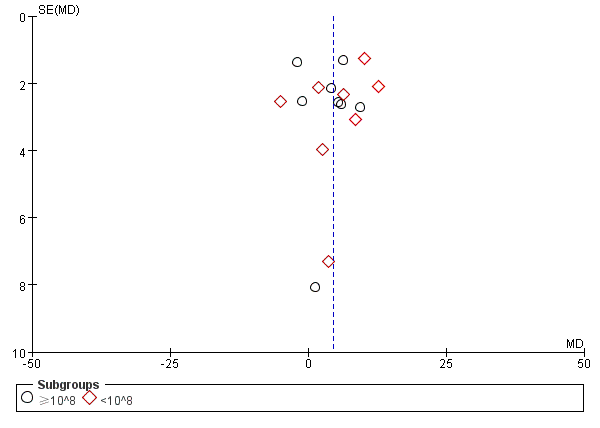


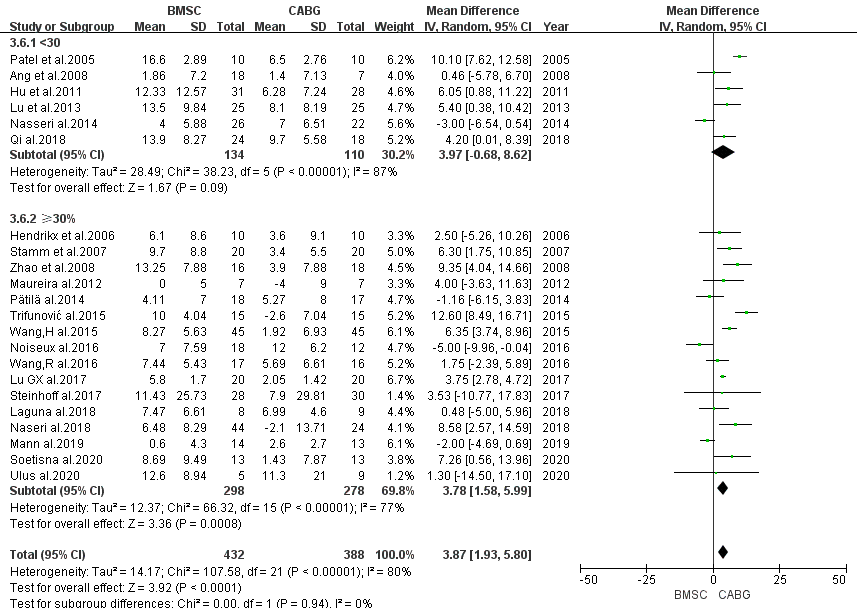

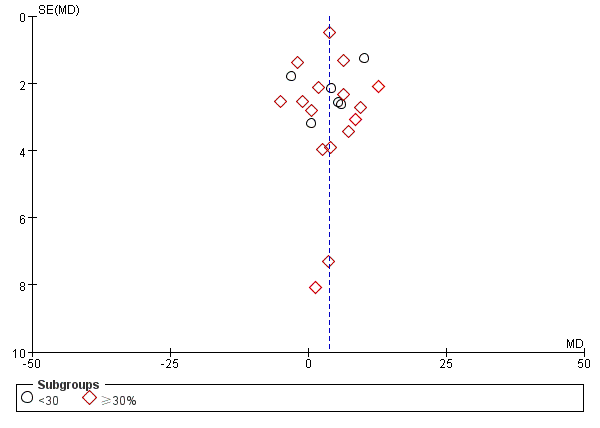

Supplement: Supplementary file 5 — Additional file 5: Subgroup analysis of LVEF (Forest plot and Funnel plot). [file 13019_2022_1838_MOESM5_ESM.docx]
